# Left Ventricular Dysfunction and Plasmatic NT-proBNP Are Associated with Adverse Evolution in Respiratory Syncytial Virus Bronchiolitis

**DOI:** 10.3390/diagnostics9030085

**Published:** 2019-07-27

**Authors:** Moises Rodriguez-Gonzalez, Alvaro Antonio Perez-Reviriego, Ana Castellano-Martinez, Simon Lubian-Lopez, Isabel Benavente-Fernandez

**Affiliations:** 1 Paediatric Cardiology Division, Puerta del Mar University Hospital, 11009 Cadiz, Spain; 2 Biomedical Research and Innovation Institute of Cadiz (INiBICA), Research Unit, Puerta del Mar University Hospital, University of Cadiz, 11009 Cadiz, Spain; 3 Paediatric Nephrology Division, Puerta del Mar University Hospital, 11009 Cadiz, Spain; 4 Neonatology Division, Puerta del Mar University Hospital, 11009 Cadiz, Spain

**Keywords:** respiratory syncytial virus, NT-proBNP, echocardiography, pulmonary hypertension, myocardial dysfunction, tissue doppler imaging, Tei index, biomarkers, infants

## Abstract

Aim: To investigate whether the presence of left ventricular myocardial dysfunction (LVMD) assessed by Tei index (LVTX) impacts the outcomes of healthy infants with Respiratory Syncytial Virus Bronchiolitis (RSVB). To explore whether N-terminal pro-B-type natriuretic peptide (NT-proBNP) increases the accuracy of traditional clinical markers in predicting the outcomes. Methods: A single-centre, prospective, cohort study including healthy infants aged 1–12 months old admitted for RSVB between 1 October 2016 and 1 April 2017. All patients underwent clinical, laboratory and echocardiographic evaluation within 24 h of admission. Paediatric intensive care unit (PICU) admission was defined as severe disease. Results: We enrolled 50 cases of RSVB (median age of 2 (1–6.5) months; 40% female) and 50 age-matched controls. We observed higher values of LVTX in infants with RSVB than in controls (0.42 vs. 0.36; *p* = 0.008). Up to nine (18%) children presented with LVMD (LVTX > 0.5), with a higher incidence of PICU admission (89% vs. 5%; *p* < 0.001). The diagnostic performance of NT-proBNP in predicting LVMD was high (area under the receiver operator characteristic curve (AUC) 0.95, CI 95% 0.90–1). The diagnostic yield of the predictive model for PICU admission that included NT-proBNP was excellent (AUC 0.945, CI 95% 0.880–1), and significantly higher than the model without NT-proBNP (*p* = 0.026). Conclusions: LVMD could be present in healthy infants with RSVB who develop severe disease. NT-proBNP seems to improve traditional clinical markers for outcomes.

## 1. Introduction

Respiratory Syncytial Virus Bronchiolitis (RSVB) is the leading cause of lower respiratory infection and hospital admission among children up to 2 years of age worldwide [1]. Approximately 2–6% cases of RSVB will develop a severe form of the disease, requiring admission to the paediatric intensive care unit (PICU) and mechanical ventilation (MV) [1,2]. RSVB constitutes approximately 13% of all PICU admissions [2]. Current guidelines recognize the identification of specific risk factors (congenital heart disease (CHD), chronic lung disease (CLD), prematurity, etc.) and clinical evaluation as the best tools to assess severity, predict evolution and tailor management [3].

Cardiovascular involvement seems to be a relevant prognostic factor in RSVB. Cardiovascular complications are present in up to 9% of cases of RSVB and constitute the second most common extrapulmonary manifestation after infectious complications [4]. These events usually present in an abrupt and unexpected manner in children with severe RSVB, and infants with CHD are particularly susceptible to these complications and adverse outcomes [5]. Interestingly, nearly half of the children admitted to PICU with RSVB are healthy prior to the clinical event [2]. In these patients, the presence of acute lung injury secondary to RSVB can also lead to important cardiovascular effects, especially elevated pulmonary vascular resistance and overload on the right ventricle (RV) [6,7,8]. Moreover, previous studies assessing the plasma levels of cardiac troponin in RSVB suggest an underrecognized yet clinically significant incidence of myocardial damage in this population [9,10,11]. Furthermore, RV global dysfunction in ventilated healthy infants has been reported [12]. Recently, we found that mild to moderate forms of pulmonary hypertension (PH) could impact the outcome of healthy infants with RSVB [13].

Adverse RV–LV interactions and left ventricle (LV) myocardial dysfunction (LVMD) are emerging as important determinants of PH outcomes. PH can induce complex changes in LV geometry and causes an abnormal relaxation and non-uniform contraction pattern in the LV wall, leading to LVMD [14,15,16]. However, most studies in healthy infants with RSVB found no abnormalities when assessing LVMD through conventional echocardiographic parameters [12,13,17,18,19]. Remarkably, there are no studies to date assessing LVMD in RSVB by more sensitive methods such as tissue Doppler imaging (TDI) echocardiography. 

N-terminal pro-B-type natriuretic peptide (NT-proBNP) is a hormone synthesized and released into the circulation by ventricular myocytes in response to pressure/volume overload and an increase in myocardial wall stress [20]. Elevated serum NT-proBNP levels have been defined as a powerful biomarker in the diagnosis of PH, and both LVMD and RV myocardial dysfunction (RVMD) secondary to pulmonary diseases [21,22,23,24,25]. Of note, we recently showed how NT-proBNP could be considered an adequate biomarker for PH in previously healthy infants with RSVB [13]. 

In this study, we aimed to investigate the presence of adverse RV–LV interactions and LVMD (assessed by TDI-echocardiography) in previously healthy infants with RSVB. We hypothesized that acute PH with RV pressure overload may indeed have a direct impact on LV performance. We also hypothesized that those infants with LVMD are prone to developing a more severe form of disease. Finally, we sought to test NT-proBNP as a biomarker for LVMD and explore whether NT-proBNP increases the accuracy of traditional clinical markers in predicting the severity of the disease. 

## 2. Materials and Methods

### 2.1. Design, Settings and Study Population

This was a single-centre, prospective, cohort study including infants aged 1–12 months old admitted to the paediatric department in our institution (a tertiary university-affiliated hospital in Spain) due to RSVB (determined by a confirmed Respiratory Syncytial Virus (RSV) antigen test) between 1 October 2016 and 1 April 2017. All patients underwent clinical, laboratory and echocardiographic evaluation within 24 h of admission. We excluded patients with co-existing CHD or CLD, prematurity, those that received MV or intravenous fluid before assessment, and those with poor-quality echocardiographic images or incomplete medical records. Severe cases were screened for coinfection and, if present, were also excluded. The control group consisted of age-matched healthy infants who underwent evaluation for heart murmur at our paediatric cardiology outpatient clinic during the study period. These controls followed the same echocardiographic protocol as study patients. Our Institutional Review Board approved the study. Informed consent was obtained for all patients.

### 2.2. Clinical and Laboratory Assessment and Outcomes

The bronchiolitis score of Sant Joan de Déu (BROSJOD) [26] was used to assess severity at admission clinically. A BROSJOD score greater than 10 points is indicative of a severe clinical state. Venous pH and pCO2 were determined and respiratory acidosis (RA) was diagnosed when pH < 7.35 and pCO2 > 45 mmHg. Plasma NT-proBNP levels at admission were determined using a commercially available electrochemiluminescent immunoassay kit (ElecSys 2010, Roche Diagnostics). The primary outcome was PICU admission during hospitalization. PICU admission criteria for RSVB at our institution include the presence of: apnea, extreme bradycardia, the need for respiratory support greater than high-flow nasal cannula oxygen therapy or inotropic support.

### 2.3. Echocardiographic Assessment

**Figure 1:**
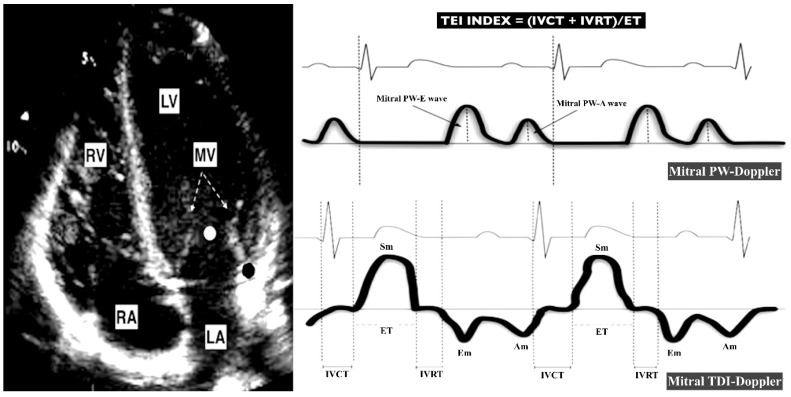
Echocardiographic parameters used for the estimation of LV performance. The left panel shows a four-chamber apical view. The right panel shows the waves used to assess LV performance. The Mitral PW-Doppler wave is obtained at the level of the LV inflow (white point). The Mitral TDI-Doppler wave is obtained at the level of the lateral mitral annulus (black point). MV (mitral valve; dotted arrow). RV (right ventricle). LV (left ventricle). RA (right atria). LA (left atria). PW (pulsed wave). DTI (Doppler tissue imaging). Sm (Doppler imaging (TDI)-derived peak systolic annular velocity). Em (TDI-derived early diastolic mitral annulus velocity). Am (TDI-derived late diastolic mitral annulus velocity). PW-E (PW-derived mitral peak early diastolic velocity). PW-A (PW-derived mitral peak late diastolic velocity). IVCT (TDI-derived isovolumic contraction time). IVRT (TDI-derived isovolumic relaxation time). ET (TDI-derived ejection time).

**Figure 2:**
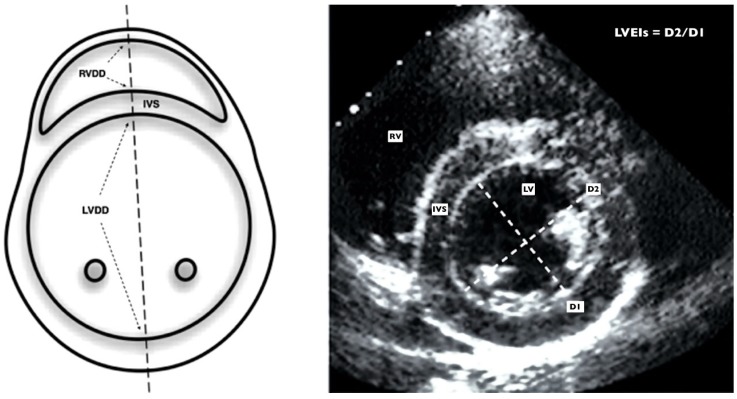
Echocardiographic parameters used for the estimation of RV and pulmonary hemodynamics. The figure shows a parasternal short axis view at the level of the papillary muscles. The left panel shows a schematic representation and the right panel shows real echocardiographic imaging. The LV systolic eccentricity index (LVEI) is obtained with de D2/D1 (dotted lines) ratio measured in systole as showed in the illustration. A LVEI > 1.2 is suggestive of raised RV pressures or pulmonary hypertension in infants^32^. RVDD (right ventricular diastolic diameter; dotted arrows). LVDD (left ventricular diastolic diameter; dotted arrows). IVS (Interventricular septum). RV (right ventricle). LV (left ventricle). D2 (LV systolic anteroposterior diameter). D1 (LV systolic laterolateral diameter).

Standard techniques to obtain M-mode, two-dimensional and Doppler (color, pulsed, continuous and TDI) echocardiograms were performed by the same experienced paediatric cardiologist (RGM) as recommended in the guidelines for the paediatric echocardiogram [27]. Images were obtained using a Phillips IE33 ultrasound scanner with an 8 or 12-MHz sectorial transducer. Each examination was recorded, and all the studies were reviewed off-line by 2 observers (RGM and PRA), who were blinded to the patient’s clinical profile. All echocardiographic measurements represent the average of 3 beats. Control and case echocardiographic data were deidentified before data analysis. Figure 1 and Figure 2 show the main echocardiographic measures used in this study.

#### 2.3.1. Right-Sided Echocardiographic Assessment

The RV end-diastolic diameter (RVEDD) and the RV/LV ratio (RVLVr) were used as indicators of RV dilation. RV systolic function was assessed by the tricuspid annular plane systolic excursion (TAPSE) [28], and by the tissue Doppler imaging (TDI) derived peak systolic annular velocity at the tricuspid level of the RV free wall (St). RV Tei index (RVTX) was used as a measurement of global (systolic and diastolic) RV function. It was calculated using the TDI-derived isovolumic contraction (IVC), isovolumic relaxation (IVR) and ejection time (ET) intervals (measured at the lateral part of the tricuspid annulus) as previously described [29]. RV systolic pressure was calculated by the systolic gradient of the tricuspid regurgitation jet (TRJG) and using the simplified Bernoulli equation [30]. Due to the absence of an adequate tricuspid regurgitation jet in most patients, the RV outflow tract acceleration time/ejection time ratio (ATET) [31], the LV systolic eccentricity index (LVEI) [32], and the presence of septal flattening at the end of systole on the qualitative assessment were also used as indicators of RV systolic pressures [30]. The LVEI was also used as a measurement of leftward displacement of the interventricular septum (IVS).

#### 2.3.2. Left-Sided Echocardiographic Assessment

The LV end-diastolic diameter (LVEDD) was used as an indicator of LV dilation. The LV shortening fraction (LVSF) and the tissue Doppler imaging (TDI)-derived peak systolic annular velocity at the mitral level of the LV free wall (Sm) were used to assess LV systolic function. To assess the diastolic LV function, we obtained the mitral peak early (E) and late (A) diastolic velocity using the pulsed wave Doppler of the mitral valve inflow. Also, the TDI-derived early diastolic mitral annulus velocity (Em) was measured at the lateral part of the mitral annulus. The E/Em ratio and E/A ratio were calculated as indicators of LV filling pressures. LV Tei index (LVTX) was used as a measurement of global (systolic and diastolic) LV function. It was calculated using the TDI-derived isovolumic contraction (IVC), isovolumic relaxation (IVR) and ejection time (ET) intervals (measured at the lateral part of the mitral annulus) as previously described [29]. Cui and Robertson reported in 2006 that the mean normal value of the LVTX for infants aged 1–12 months is 0.35 (0.09), and that a LVTX less than 0.5 is the upper limit of normal (2Zscore) [33]. Therefore, a LVTX > 0.5 was defined as LVMD in this study.

### 2.4. Reproductibility

To explore intra-observer and inter-observer agreement, 30 echocardiographic studies were randomly selected and analysed offline. To estimate the intra-observer agreement, the first observer (RGM) remeasured the LVTX, RVTX and LVEI with a 30-day interval blinded to previous measurements and patient information. To assess the inter-observer agreement, the means of both observers for each measurement were compared.

### 2.5. Statistical Analysis

Continuous data are presented as the median (range) or mean (standard deviation) after testing for normality with the Shapiro–Wilk test; categorical data are presented as frequencies and percentage. Mean comparison was performed using the Student’s *t* test or Wilcoxon Mann–Whitney test as appropriate. Proportions were compared using the Chi-square test or exact methods as necessary. Pearson and Spearman coefficients were used to assess correlations between continuous data. A receiver operator curve (ROC) analysis was used to determine the diagnostic accuracy of NT-proBNP for LVMD. The best cut-off value of NT-proBNP to detect LVMD was empirically estimated based on the Liu method, and values of sensitivity (Se), specificity (Sp), negative predictive value (NPV) and positive predictive value (PPV) were calculated for the obtained cut point. We selected 2 predefined predictive models for PICU admission. The selection of the included variables was based on the theoretical background and the exploratory analysis. Model 1 (proposed model) included age, BROSJOD score and NT-proBNP levels and model 2 (traditional model) included age and BROSJOD score. Prediction models were evaluated through multivariate logistic regression analysis. The discriminating ability of each model was assessed by the area under the receiver operator characteristic (AUC) curve. The AUCs from the obtained models were then compared by using the DeLong method [34] to determine whether any model resulted in increased predictive accuracy. The reliability of echocardiographic measurements was evaluated with the intra-class correlation (ICC) coefficients and Bland–Altman (BA) analysis [35]. Based on the 95% confident interval of the ICC coefficients, values less than 0.5, between 0.5 and 0.75, between 0.75 and 0.9, and greater than 0.90 were considered indicative of poor, moderate, good, and excellent reliability, respectively. All the statistical analyses were performed using the Stata 13.0. (StataCorp. 2013. Stata Statistical Software: Release 13. College Station, TX: StataCorp LP.). A *p* value < 0.05 was considered statistically significant.

## 3. Results

### 3.1. Baseline Characteristics and Outcomes of Patients with RSVB

We enrolled a total of 50 cases of RSVB with a median age of 2 (1–6.5) months (40% female). The control group consisted of 50 healthy infants with no differences regarding age, sex or body surface area (BSA) distribution (Table 1). RSVB patients were admitted 2.76 (1.23) days after the initial symptoms, with a median BROSJOD score of 6 (1–14), a median SpO2 of 93% (87–98%), and a median heart rate of 118 bpm (89–179 bpm). Up to nine (18%) cases presented RA and a BROSJOD score > 10. A total of 10 (20%) cases needed PICU admission within 1.20 (0.38) days from hospitalization (length of PICU stay 5 (2–9) days) and were classified as having severe RSVB. A total of 3 of these 10 cases required MV and seven of them required continuous positive airway pressure (CPAP). No cases of arrhythmia different from sinus tachycardia were observed. No patient required inotropic support and none of the included patients died.

### 3.2. Echocardiographic Alterations in Patients with RSVB

**Figure 3:**
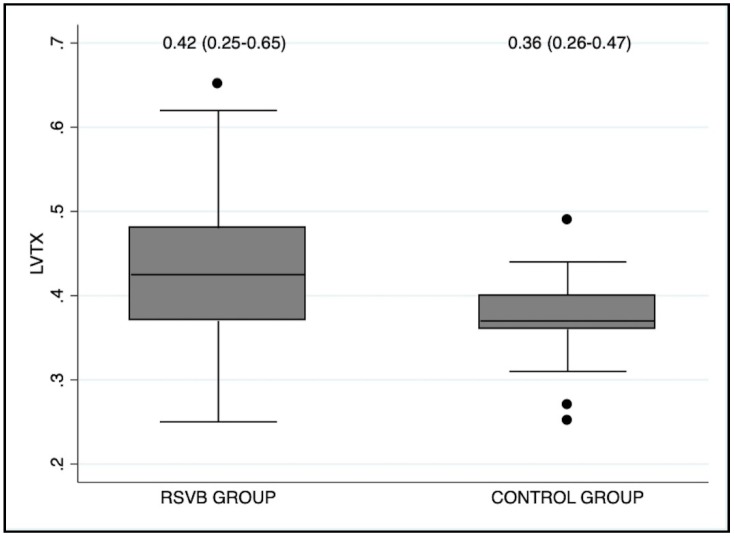
Box-plot diagram showing that the cases of RSVB presented higher values of LVTX at admission than healthy controls (*p* = 0.008). LVTX (Left ventricular Tei index).

The RSVB group had a higher proportion of pericardial effusion (34% vs. 6%; *p* < 0.001). All cases of pericardial effusion were mild and did not require any treatment. We observed higher values of LVTX (0.42 vs. 0.36; *p* = 0.008) in infants with RSVB than in controls (Figure 3). The RSVB group also presented more cases of septal flattening (28% vs. 6%; *p* = 0.003), and higher TRJG (27 vs. 22 mmHg; *p* = 0.013), RVTX (0.39 vs. 0.36; *p* = 0.005) and LVEI (1.08 vs. 1; *p* < 0.001) than the control group. There were no differences between RSVB and control groups regarding echocardiographic parameters of ventricular dimensions, systolic or diastolic function (Table 2).

### 3.3. Adverse LV–RV Interactions in Patients with RSVB

**Figure 4:**
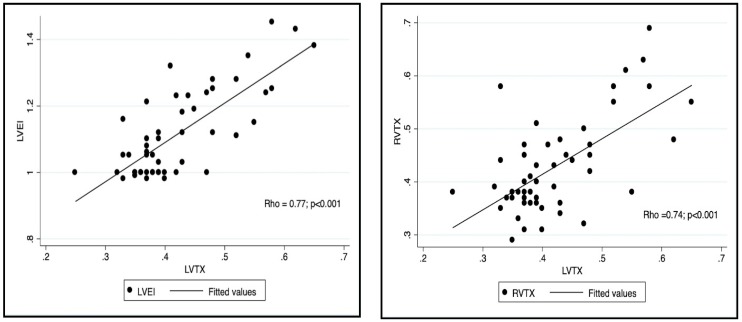
A representation of the correlation found between LVTX and parameters of RV function (RVTX, right panel) and RV pressures (LVEI, left panel). LVTX (Left ventricular Tei index). RVTX (right ventricular Tei index). LVEI (systolic left ventricular eccentricity index).

In the RSVB group, increased LVTX was related to echocardiographic parameters indicating higher RV dimensions (*Rho* RVDD = 0.56, *Rho* RVLVr = 0.60), higher RV pressures (*Rho* TRJG = 0.54, *Rho* ATET = −0.50, *Rho* LVEI = 0.77), and decreased RV global function (*Rho* RVTX= 0.74). We did not find associations of echocardiographic measurements of RV systolic function (TAPSE and St) with LVTX (Table 3 and Figure 4).

### 3.4. LVMD in Patients with RSVB

**Figure 5:**
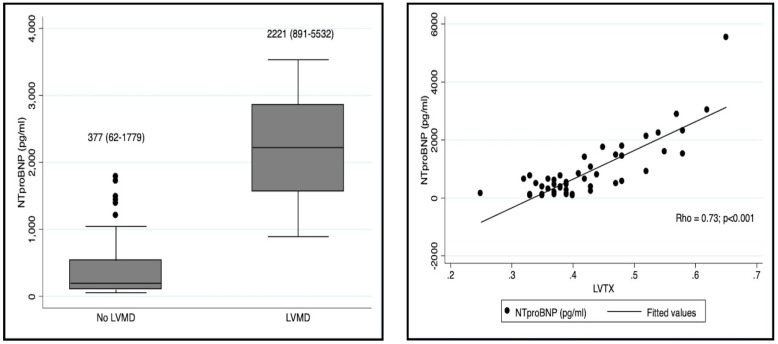
The left panel is a box-plot diagram representing the comparison of NT-proBNP levels between patients with and without LVMD. The right panel represents the correlation found between LVTX and NT-proBNP levels. LVTX (Left ventricular Tei index); LVMD (Left ventricular myocardial dysfunction).

We found LVMD, defined by a LVTX > 0.5, in nine (18%) patients with RSVB (Table 4). LVMD was associated with PICU admission (89% vs. 5%; *p* < 0.001). These patients presented with a higher BROSJOD score (11 vs. 6; *p* < 0.001), lower SpO2 (90% vs. 94%; *p* < 0.001), more cases of RA (55% vs. 9%; *p* = 0.001), and higher NT-proBNP levels (2221 pg/mL vs. 377 pg ml; *p* < 0.001) than those with a normal LV myocardial function. The LVTX was also strongly correlated with NT-proBNP levels (*Rho* = 0.73) (Figure 5).

### 3.5. NT-ProBNP as Biomarker for LVMD

**Figure 6:**
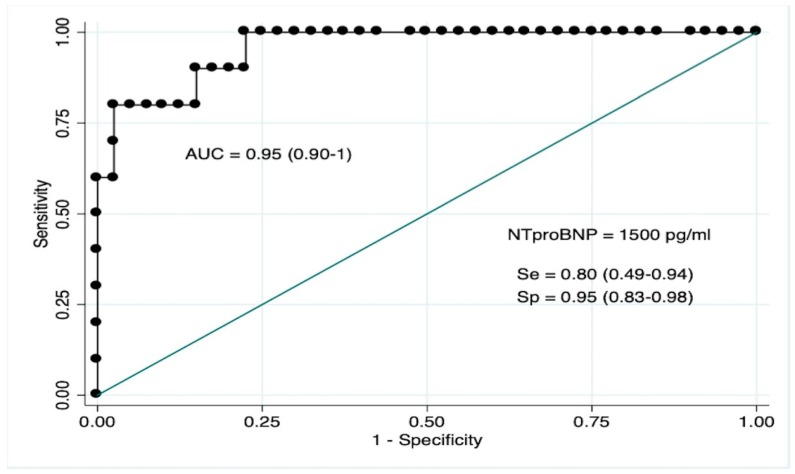
Representation of the receiver operating characteristic curve of NT-proBNP to detect LVMD in infants with RSVB. LVMD (Left ventricular myocardial dysfunction).

The diagnostic performance of NT-proBNP to predict LVMD in infants with RSVB resulted in an area under the ROC curve of 0.91 (CI95% 0.79–0.98) (Figure 6). The best estimated cut-off value to predict LVMD on echocardiography was 1500 pg/mL, correctly classifying 92% of cases, with a Se of 0.80 (CI95% 0.49–0.94), Sp of 0.95 (CI95% 0.83–0.98), a PPV of 0.80 (CI95% 0.49–0.94), and an NPV of 0.95 (CI95% 0.87–0.99) (Youden index 0.75).

### 3.6. Clinical and Laboratory Predictors of PICU Admission in RSVB

**Figure 7:**
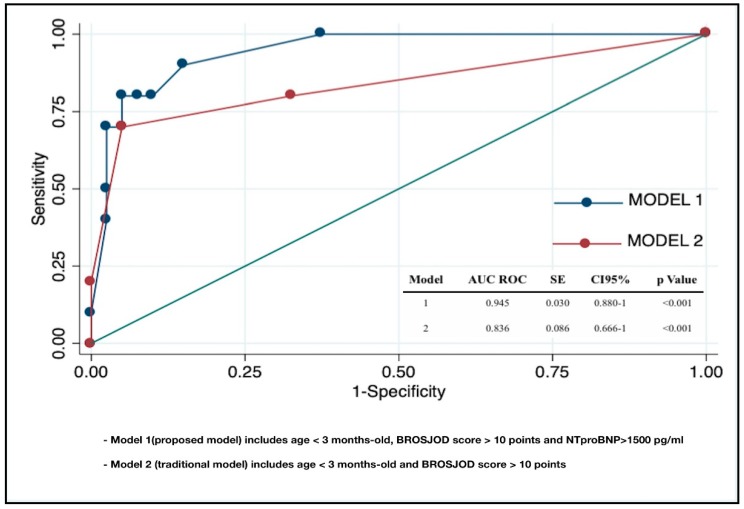
Graphical representation of the comparison between the areas under the receiver operator characteristic curves of the predictive models selected.

We developed two different and predefined prediction models for PICU admission in RSVB. The variables used were clinical parameters that are traditionally used to assess severity in RSVB (age and clinical score), and NT-proBNP as biomarker for LVMD. Model 1 (the proposed model) included age < 3 months, BROSJOD score > 10, and NT-proBNP > 1500 pg/mL. Model 2 (the traditional model) included age < 3 months and BROSJOD score > 10. The diagnostic yield of model 1 for PICU admission was excellent (AUC 0.945, CI95% 0.880–1), and significantly higher than the yield for model 2 (*p* = 0.026). In model 1, the presence of NT-proBNP levels > 1500 pg/mL was the only independent predictive factor for PICU admission in RSVB, with an OR 27.03 (CI95% (1.50–487), *p* = 0.025) (Table 5 and Figure 7).

### 3.7. Reproductibility

The intra-observer and inter-observer agreement for LVTX, RVTX and LVEI were good or excellent, with all ICC coefficients > 0.75 (Table 6).

## 4. Discussion

The main finding of our study is that LVMD was observed at the early stages of the disease in up to 18% of previously healthy infants with RSVB when assessed by DTI-derived LVTX. The LVMD was associated with a more severe respiratory state, PICU admission, and echocardiographic signs of RV pressure overload and RVMD, indicating the presence of adverse RV–LV interactions in cases of severe RSVB. Also, we observed that NT-proBNP accurately identified LVMD. Moreover, we found an added benefit to the addition of NT-proBNP to the clinical evaluation in predicting the development of severe disease in this population.

CHD is an important cause of morbidity and mortality in RSVB [5]. This may be related to multiple physiological factors including baseline compromised cardiorespiratory function and the potential development of PH. However, little is known about LVMD and its association with RV function, pulmonary hemodynamics, and outcomes in previously healthy infants with RSVB. In accordance with the literature, the present study reveals that conventional parameters of myocardial function are not altered in RSVB [17,18,19]. Only one previous study has assessed myocardial performance using the TEI index in RSVB [12]. Our results are consistent with those reported by Thorburn et al. who found RVMD in ventilated patients with severe RSVB. However, they did not demonstrate any association between PH and RVMD. This may be due to the use of TRJG alone as an echocardiographic marker of RV pressure, and the presence of PH may had been underestimated. In a recent work from our group, we used a combination of different echocardiographic parameters to assess RV pressures and PH was reported in up to 22% of RSVB cases at early stages of the disease. In agreement with Thorburn et al., we did not find an association between PH and RV or LVMD [13]. We assessed ventricular function only by conventional parameters (TAPSE and LVSF).

To the best of our knowledge, this is the first study to evaluate LVMD using TDI-derived LVTX, and to assess RV–LV interactions in infants with RSVB. LVTX, which includes both systolic and diastolic time intervals to assess the global cardiac dysfunction, is an easily performable, recordable and reproducible parameter with normal reference values that can be applied to the entire spectrum of the paediatric population, regardless of age, heart rate, and BSA [36]. Using LVTX, we found LVMD in nearly 20% of cases. The RV shares muscle fibres, the inter-ventricular septum (IVS), and the pericardial sac with the LV. Consequently, changes in RV affect the LV, a concept termed ventricular interdependence [16,37,38]. In this study, we observed a moderate to strong correlation between LVTX and leftward displacement of the IVS (LVEI), raised RV pressures (TRGJ, ATET) and reduced RV global function (RVTX), confirming the presence of adverse RV–LV interactions in cases with severe RSVB. These results do not imply casualty, but in the absence of primary (myocarditis, cardiomyopathies, CHD) or secondary (sepsis, severe acidosis...) causes of LVMD, we suggest RV pressure overload and RV dysfunction due to pulmonary disease as the underlying condition for LVMD in our RSVB cohort. Recent paediatric studies have reported that PH can induce complex changes in LV geometry and causes an abnormal relaxation and non-uniform contraction pattern in the LV wall, leading to LVMD [14,15,16], supporting our hypothesis. These observations could add new insights into the pathophysiology of RSVB, highlighting a key role of the cardiovascular system, especially LV myocardial performance in this setting. Validating our findings in similar populations in different settings may also provide a basis to implement new therapeutic approaches for this disease, which currently has no effective treatment. Possible new therapeutic approaches may include the initiation of early respiratory support in cases with increased NT-proBNP, pulmonary vasodilators to reduce RV pressure overload and avoiding epinephrine in cases with LVMD.

In RSVB, the major goals are the prevention and early identification of infants at risk for severe disease in order to provide the best management options and decrease morbidity. Current guidelines recommend only clinical observation for this purpose in infants without known comorbidities [3]. However, most clinical scores for RSVB are not well validated and fail to predict outcomes [39,40]. Recently, the BROSJOD score, a validated clinical score for RSVB, has shown a strong capacity to predict the evolution in the course of RSVB, but it is not yet generalizable due to the single-centre character of the study [26]. In this context, the identification of novel biomarkers with adequate predictive value for disease severity in RSVB is an area of increasing research interest. Neutrophins, cytokines and leukotrienes are promising but not widely available for clinical practice [41]. Previous studies have also tested cardiac troponin as a prognostic marker with inconsistent results [9,10,12,13]. The LVMD found in our population was only identifiable by TDI-echocardiography, suggesting that it was mild in most of our patients. Nevertheless, this does not mean that LVMD is inconsequential in RSVB. Remarkably, most patients with LVMD presented with severe disease at admission and most of them required PICU admission. Of note, we included non-ventilated infants at early stages of the disease (mean time of 2.76 (1.23) days after the initial symptoms), when the patients had not yet been admitted to the PICU, increasing the prognostic value of our results. Therefore, assessing and understanding myocardial function in RSVB seems to be relevant.

Another interesting finding of our study was that NT-proBNP could be a useful biomarker for LVMD and subsequent outcomes in RSVB. Previous studies have also documented the correlation between LVTX and NT-proBNP plasma levels [42,43,44]. We explored the diagnostic accuracy of NT-proBNP to detect LVTX > 0.50, which was high (AUC 0.91), with an optimal cut-off value of 1500 pg/mL (Se 0.80, Sp 0.95, PPV 0.80, NPV 0.95). We also tested the benefit in adding NT-proBNP to the currently used clinical data to assess outcomes in RSVB. Although the predictive models including age and BROSJOD score presented a high predictive accuracy for PICU admission in our population, we observed that the addition of NT-proBNP to this model increased the predictive value significantly, and that NT-proBNP was the only independent factor within the analysed values that predicted a severe course of the disease. Most cases of RSVB are mild forms manageable on an outpatient basis without the need for laboratory exams. Nevertheless, many children sufficiently ill to require hospitalization will routinely have laboratory studies drawn. Adding NT-proBNP measurement to these studies could be useful in order to identify high-risk patients who benefit from echocardiographic screening to PH, RVMD or LVMD. Based on our results, it might be reasonable to perform an echocardiogram in those patients with NT-proBNP levels > 1500 pg/mL.

This work included some limitations. Our study was performed at a single centre and with a relatively small size. We excluded irritable or unstable patients, where the technical difficulties due to respiratory comorbidity and the patient’s inability to tolerate the evaluation could impact the results. The PICU admission criteria and, subsequently, the definition of severe diseases in the study are based on the protocol of our hospital, which can vary between institutions. Therefore, a larger multicentre cohort study including irritable or unstable cases and with uniform PICU admission criteria may be needed for the verification and generalization of our results. Finally, the assessment of myocardial function and PH on echocardiography was not confirmed by an independent gold-standard method, such as cardiac magnetic resonance imaging or right heart catheterization, and therefore some patients could have been misclassified.

## 5. Conclusions

Adverse RV–LV interactions and LVMD could be present in healthy infants with RSVB during the early stages of the disease, negatively impacting the outcome. NT-proBNP seems to be an adequate biomarker for LVMD. Adding NT-proBNP to traditional clinical markers to assess outcomes in RSVB could improve the early detection of those cases that will develop a severe illness. Future research is need in order to confirm these results and to design new therapeutic approaches based on them.

## Figures and Tables

**Table 1:** Baseline clinical and laboratory characteristics of the Respiratory Syncytial Virus Bronchiolitis (RSVB) population and comparison with controls.

Variable	RSVB Group (*n* = 50)	Control Group (*n* = 50)	*p* Value
Age (months)	2 (1–6.5)	2 (1–9)	0.591
Female sex	20 (40)	23 (46)	0.545
BSA (m2)	0.28 (0.18–0.43)	0.27 (0.18–0.42)	0.617
Time of symptoms (days)	2.76 (1.23)	-	-
BROSJOD score	6 (1–14)	-	-
SpO2 (%)	93 (87–98)	99 (95–100)	<0.001
Heart rate (bpm)	118 (89–179)	109 (87–133)	0.002
pH	7.36 (7.22–7.45)	-	-
pCO2	42 (31–71)	-	-
Nt-proBNP (pg/mL)	511 (62–5532)	-	-
Respiratory Acidosis	9 (18)	-	-
BROSJOD > 10	9 (18)	-	-
PICU admission	10 (20)	-	-

BSA (body surface area); PICU (paediatric intensive care unit); BROSJOD score (bronchiolitis score of Sant Joan de Déu); Nt-proBNP (N-terminal pro-B-type natriuretic peptide); PICU (Paediatric intensive care unit).

**Table 2:** Baseline echocardiographic characteristics of the RSVB population and comparison with controls.

Variable	RSVB Group (*n* = 50)	Control Group (*n* = 50)	*p* Value
Pericardial effusion	17 (34)	3 (6)	<0.001
**Left ventricle**			
LVDD (mm)	21 (17–29)	21 (14–28)	0.470
LVSF (%)	38.5 (5.6)	39 (5)	0.851
Sm (cm/s)	8.7 (1.6)	9 (1.2)	0.573
Mitral E (cm/s)	98 (79–125)	96 (76–123)	0.798
Mitral A (cm/s)	75 (49–97)	71 (51–93)	0.259
Mitral E/A	1.25 (0.93–1.98)	1.43 (1–2)	0.200
Mitral Em (cm/s)	9.4 (6–5–14)	10 (6.6–15)	0.110
Mitral Am (cm/s)	9 (5–15)	9 (6–13)	0.182
Mitral E/Em (cm/s)	10.2 (5.8–20)	9.8 (5.2–17)	0.283
LVTX	0.42 (0.25–0.65)	0.36 (0.26–0.47)	0.008
**Right ventricle**			
RVDD (mm)	10 (7–19)	10 (7–14)	0.264
RV/LV ratio	0.49 (0.28–0.80)	0.46 (0.28–0.76)	0.473
TAPSE (mm)	12.3 (1.6)	12 (1.8)	0.624
St (cm/s)	8.5 (2)	9.1 (1.7)	0.901
RVTX	0.39 (0.25–0.65)	0.37 (0.26–0.48)	0.005
Adequate TRJ	25 (50)	30 (60)	0.633
TRJG (mmHg)	27 (18–47)	22 (16–34)	0.013
ATET	0.38 (0.06)	0.39 (0.03)	0.288
LVEI	1.08 (0.98–1.45)	1 (0.95–1.12)	<0.001
Septal Flattening	14 (28)	3 (6)	0.003

LVDD (left ventricle diastolic diameter); LVSF (left ventricular shortening fraction); LVTX (left ventricular Tei index); RVDD (right ventricular diastolic diameter); RV (right ventricle); LV (Left ventricle); TAPSE (tricuspid annular plane systolic excursion); RVTX (Right ventricular Tei index), TRJ (tricuspid regurgitation jet); TRJG (tricuspid regurgitation jet gradient); ATET (right ventricular acceleration time/right ventricular ejection time ratio); LVEI (systolic left ventricular eccentricity index).

**Table 3:** Correlation coefficients between LVTX and RV echocardiographic parameters and plasmatic NT-proBNP levels in RSVB cases.

Variable	Rho	*p* Value
RVDD	0.56	0.003
RV/LV ratio	0.60	0.001
TAPSE	−0.19	0.159
St	−0.12	0.217
RVTX	0.738	<0.001
TRJG	0.54	0.004
ATET	−0.50	0.009
LVEI	0.77	<0.001
NT-proBNP	0.73	<0.001

RVDD (right ventricular diastolic diameter); RV (right ventricle); LV (Left ventricle); TAPSE (tricuspid annular plane systolic excursion); RVTX (Right ventricular Tei index); TRJ (tricuspid regurgitation jet); TRJG (tricuspid regurgitation jet gradient); ATET (right ventricular acceleration time/right ventricular ejection time ratio); LVEI (systolic left ventricular eccentricity index).

**Table 4:** Clinical and laboratory characteristics in patients with RSVB and LVMD, and comparison with those patients with normal LV function.

Variable	LVMD (*n* = 9; 18%)	Normal LV Function (*n* = 41; 82%)	*p* Value
Age (months)	2 (1–4.5)	2 (1–6.5)	0.538
Female sex	2 (22)	18 (44)	0.230
BSA (m^2^)	0.29 (0.18–0.33)	0.28 (0.18–0.43)	0.742
Time of symptoms (days)	2 (1.5)	3 (1–7)	0.488
BROSJOD score	11 (9–14)	6 (1–13)	<0.001
SpO2 (%)	90 (87–93)	94 (88–98)	<0.001
Heart rate (bpm)	135 (100–164)	116 (89–179)	0.141
pH	7.3 (7.25–7.42)	7.36 (7.22–7.45)	0.115
pCO2 (mmHg)	54 (31–61)	41 (31–71)	0.009
Nt-proBNP (pg/mL)	2221 (891–5532)	377 (62–1779)	<0.001
PICU admission	8 (89)	2 (5)	<0.001

LVMD (left ventricular myocardial dysfunction); BSA (Body surface area); PICU (paediatric intensive care unit).

**Table 5:** Univariate and Multivariate logistic regression analysis performed to find a predictive model for PICU admission during hospitalization in our RSVB cohort.

Variable	Univariate Analysis OR (CI 95%)	*p* Value	Multivariate Analysis OR (CI 95%)	*p* Value	pseudoR2
**Model 1**				<0.001	0.51
Age < 3 months	0.88 (0.19–4.04)	0.875	0.71 (0.66–7.58)	0.777	
BROSJOD score > 10	44.33 (6.22–315.50)	<0.001	3.06 (0.12–76.44)	0.494	
NT-proBNP > 1500 pg/mL	76 (9.27–622)	<0.001	27.03 (1.50–487)	0.025	
**Model 2**				<0.001	0.38
Age < 3 months	0.88 (0.19–4.04)	0.875	0.52 (0.06–4.21)	0.541	
BROSJOD score > 10	44.33 (6.22–315.50)	<0.001	49.27 (6.38–380)	<0.001	

OR (Odds ratio); CI (Confidence interval).

**Table 6:** Intra-observer and inter-observer agreement scores for the main echocardiographic measurements performed in this study, the left ventricular and right ventricular Tei indexes (LVTX and RVTX), and the systolic left ventricular eccentricity index (LVEI).

Variable	Intra-observer (RG.M)		Inter-observer (RG.M and PR.A)	
	ICC (95% CI)	BA (LOA)	ICC (95% CI)	BA (LOA)
LVTX	0.95 (0.93–0.99)	0.00 (−0.03–0.03)	0.84 (0.74–0.93)	−0.01 (−0.07–0.05)
RVTX	0.97 (0.95–0.99)	−0.002 (−0.02–0.02)	0.86 (0.77–0.95)	−0.015 (−0.07–0.04)
LVEI	0.89 (0.82–0.96)	−0.007 (−0.04–0.03)	0.84 (0.73–0.94)	−0.01 (−0.05–0.03)

ICC (interclass coefficient); CI (confidence interval); BA (Bland–Altman average); LOA (limits of agreement).
